# Proton pump inhibitors use is associated with a higher prevalence of kidney stones: NHANES 2007–2018

**DOI:** 10.1186/s12889-024-18710-8

**Published:** 2024-05-02

**Authors:** Youjie Zhang, Minghui Liu, Zewu Zhu, Hequn Chen

**Affiliations:** 1 Department of Urology, Xiangya Hospital, Central South University, Changsha, 410008 China; 2 National Clinical Research Center for Geriatric Disorders, Xiangya Hospital, Central South University, Changsha, China

**Keywords:** Proton pump inhibitors, Kidney stones, National Health and Nutrition Examination Survey, Calcium oxalate

## Abstract

**Background:**

Proton pump inhibitors (PPIs) are widely used throughout the world as an effective gastrointestinal drug. Nevertheless, according to the existing literature, PPIs can reduce the excretion of magnesium, calcium and other components in urine, which may promote the formation of kidney stones. We used the National Health and Nutrition Examination Survey (NHANES) database to further investigate the association between the use of PPIs and the prevalence of kidney stones.

**Methods:**

We performed a cross-sectional analysis using data from 2007 to 2018 NHANES. PPIs use information of 29,910 participants was obtained by using prescription medications in the preceding month, and kidney stones were presented by a standard questionnaire. Multiple regression analysis and stratified analysis were used to estimate the association between PPIs use and kidney stones after an adjustment for potential confounders.

**Results:**

The multiple logistic regression indicated that the PPIs exposure group (P1) had a significantly higher risk of nephrolithiasis than the PPIs non-exposure group (P0) in Model 3 (OR 1.24, 95% CI 1.10–1.39, *P* < 0.001). The stratified analyses indicated there were significant statistical differences between PPIs use and kidney stones among females (OR 1.36, 95% CI 1.15–1.62, *P* < 0.001), non-Hispanic whites (OR 1.27, 95% CI 1.09–1.48, *P* = 0.002), individuals with an education level than 11th grade (OR 1.41, 95% CI 1.13–1.76, *P* = 0.002) and individuals with an annual family income of $0 to $19,999 (OR 1.32, 95% CI 1.06–1.65, *P* = 0.014) and $20,000 to $44,999 (OR 1.25, 95% CI 1.02–1.54, *P* = 0.033) in Model 3.

**Conclusions:**

Our study revealed that PPIs use is associated with a higher prevalence of kidney stones for the US population, primarily among women, non-Hispanic whites, individuals with low education levels and individuals with low household income levels. Further studies are required to confirm our findings.

**Supplementary Information:**

The online version contains supplementary material available at 10.1186/s12889-024-18710-8.

## Background

Nephrolithiasis is a prevalent ailment that affects one out of every eleven persons in the United States [1]. Nephrolithiasis incidence has risen dramatically during the last three decades, putting an even greater financial strain on patients [1, 2]. Approximately 80% of kidney stones consist mainly of calcium oxalate (CaOx), many of which grow on Randall’s plaque (RP) on the surface of the renal papilla [3]. There are many risk factors for kidney stones, such as hypertension, diabetes, obesity, etc [3–5]. . Dietary choices and lifestyle are also important, and calcium and hydration consumption are inextricably linked [6, 7].

Proton pump inhibitors (PPIs) are widely used worldwide. PPIs can reduce gastric acid secretion and are often used to treat gastroesophageal reflux disease (GERD), Helicobacter pylori infection and peptic ulcer disease (PUD) [8–10]. Although PPIs have powerful curative effects, they are often associated with inappropriate use, such as overuse [11]. At the same time, we should not ignore the adverse reactions caused by PPIs, such as enteric infection, kidney disease, a higher risk of hip fracture, and changes in the structure of the stomach [12–14].

PPIs can reduce the excretion of magnesium, calcium and citrate in the urine [15–17]. Furthermore, the intestinal absorption of calcium is lower as a result of PPIs inhibiting gastric acid production [18–20]. The decrease in calcium absorption and urinary calcium excretion has a promoting effect on reducing the formation of kidney stones [21, 22]. However, the decrease in urinary magnesium and urinary citrate excretion caused by PPIs will increase the risk of kidney stones [16, 17, 22–24]. Based on existing research results, we explored the relationship between PPIs and the prevalence of kidney stones from the data in the NHANES database.

## Materials and methods

### Study population

**Fig. 1:**
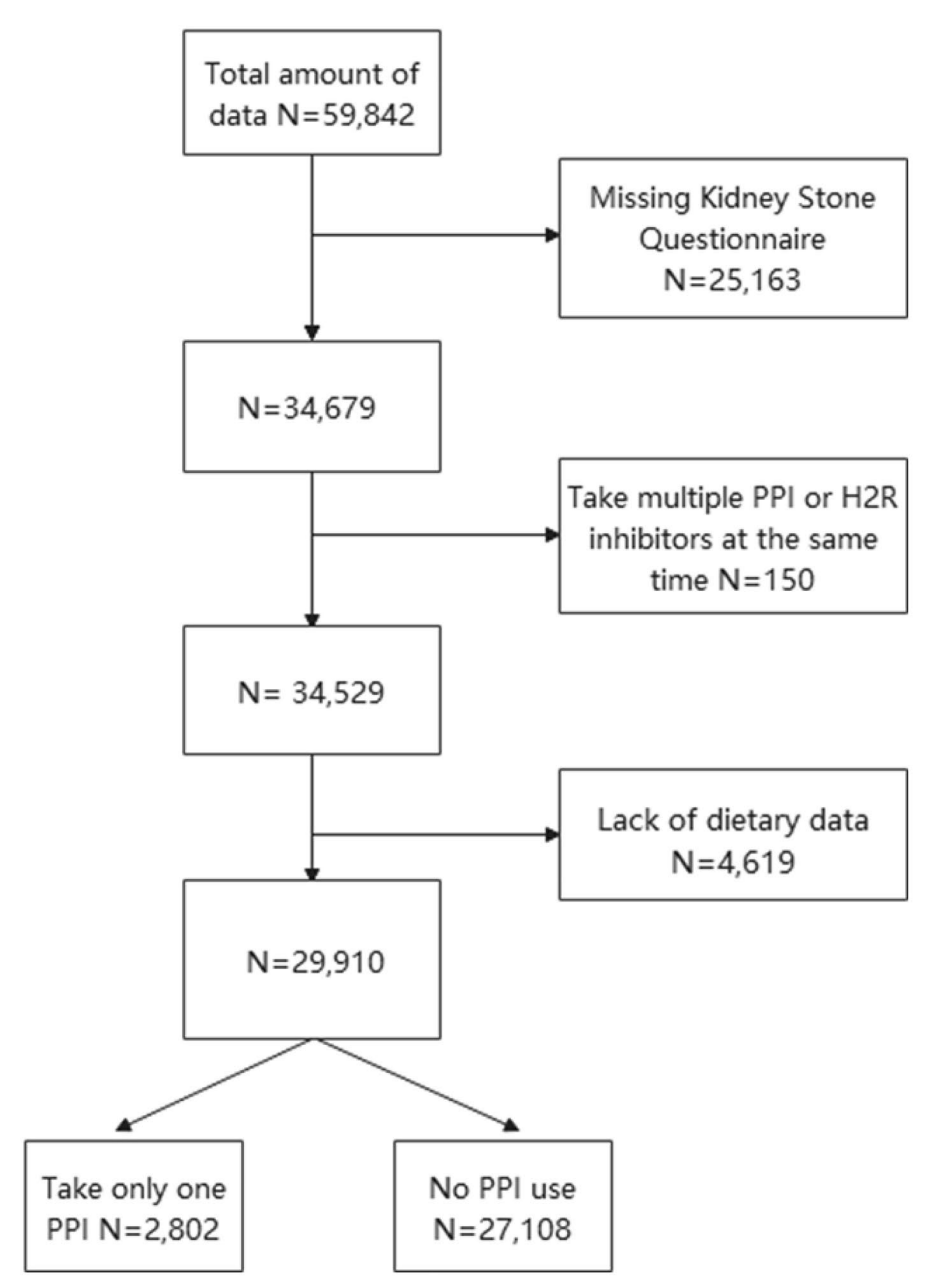
Schematic diagram on the selection of the study participants

The data were derived from six consecutive cycles of NHANES conducted between 2007 and 2018. There were 59,842 participants aged 18–80 years in NHANES 2007–2018. The exclusion criteria were as follows: (a) missing kidney stone questionnaire (*n* = 25,163); (b) take multiple PPIs or H2R inhibitors at the same time (*n* = 150); (c) lack of dietary data (*n* = 4,619); A total of 29,910 participants participated in the study (Fig. 1).

### Study variables

The independent variable in this study is the use of PPIs. We obtained the types and duration of PPIs use from the prescription medications questionnaires. The PPIs categories include omeprazole, esomeprazole, lansoprazole, dexlansoprazole, pantoprazole and rabeprazole. The dependent variable is the formation of kidney stones.

We included the following covariates based on the previous studies on dietary intake and nephrolithiasis [25–28]: age, marital status (married and unmarried), gender (male/female), race (Mexican American, other Hispanic, Non-Hispanic white, Non- Hispanic black and other), BMI (< 25.0 kg/m^2^ and ≥ 25.0 kg/m^2^), education level (less than 11th grade, high school or equivalent, some college or AA degree, and college graduate or above), vigorous and moderate recreational activities, annual family income ($0–$19,999, $20,000–$44,999, $45,000–$74,999, ≥$75,000 and other), hypertension, diabetes, daily intake of total energy, water, protein, calcium, phosphate, potassium, sodium, magnesium, zinc, alpha-carotene, beta-carotene, caffeine, alcohol, and vitamins A, B6, C, D, E and K. The diagnostic criteria for diabetes are as follows: fasting blood glucose level should be equal to or greater than 7.0 mmol/L, or two-hour blood glucose level must be equal to or greater than 11.1 mmol/L during 75 g oral glucose tolerance test (OGTT), or A1C level should be greater than or equal to 6.5%. Additionally, the diagnostic criteria for borderline diabetes, also known as prediabetes, are as follows: fasting blood glucose level should be between 5.6 and 6.9 mmol/L, or two-hour blood glucose level during 75 g OGTT should be between 7.8 and 11.0 mmol/L, or A1C level should be between 5.7 and 6.4% [29].

We extracted personal interview data about kidney stones from participants aged 20 and above from the NHANES 2007–2018 (Kidney Conditions - Urology). The history of kidney stones is judged by the answer “Have you ever had kidney stones?“(KIQ026).

### Statistical analysis

We described the data as the mean ± standard error (SE) for continuous variables and the percentage (%) for categorical variables. The Kruskal Wallis test was used to evaluate continuous variables, and the Chi-square (χ2) test was used to analyze categorical variables. Three different weighted logistic regression models were used to calculate the odds ratio (OR) and 95% confidence interval (CI) of PPIs usage to kidney stones. The weights which were selected for data analysis to represent US population referenced the instructions provided by the NHANES database (https://wwwn.cdc.gov/nchs/nhanes/tutorials/module3.aspx). We applied mobile examination center (MEC) exam weight (WTMEC2YR) for all analysis, as described by a previous study [30]. Additionally, we conducted sub-analysis stratified by gender, race, education, and annual family income. We adjusted nothing in Model 1 and adjusted age, gender, and race in Model 2. Model 3 were further adjusted for marital status, education level, vigorous and moderate recreational physical activities, annual family income, hypertension, diabetes, BMI, energy, water, dietary intakes of calcium, phosphate, sodium, potassium, magnesium, zinc, Alpha-carotene, Beta-carotene, dietary fiber, caffeine, alcohol, vitamins A, B6, C, D, E, and K. Effect sizes with 95% confidence intervals (CIs) were displayed. Two-tailed *P* values < 0.05 were considered as a statistically significant difference. An interactive feature has been added to enable the examination of correlations between different groups.

All statistical analysis was performed using the software Empower Stats (http://www.empowerstats.com) and R package (http://www.R-project.org).

## Results

### Participant characteristics

According to our inclusion and exclusion criteria, we extracted 29,910 participants’ data from NHANES 2007–2018, of which 2,802 had kidney stones and 27,108 had not. Characteristics of participants are presented as two groups in Table 1. There are significant statistical differences in the following variables, including PPIs usage (*P* < 0.001), age (*P* < 0.001), gender (*P* < 0.001), race (*P* < 0.001), marital status (*P* < 0.001), vigorous recreational activities (*P* < 0.001), moderate recreational activities (*P* < 0.001), education level (*P* < 0.001), hypertension (*P* < 0.001), diabetes (*P* < 0.001), protein (*P* = 0.003), dietary fiber (*P* = 0.018), phosphorus (*P* = 0.015), magnesium (*P* < 0.001), caffeine (*P* < 0.001), Alcohol (*P* < 0.001), Moisture (*P* = 0.038), Vitamin B6 (*P* = 0.002), Vitamin C (*P* < 0.001), alpha-carotene (*P* = 0.027) and beta-carotene (*P* < 0.001). Those with kidney stones were more likely to be male, non-Hispanic white, married, hypertension-positive, diabetes-positive, some college or AA degree. They were less likely to take vigorous recreational activities and moderate recreational activities.

**Table 1:** Characteristics of participants in NHANES 2007–2018

Characteristic	None-stone formers No. (%)	Stone formers No. (%)	*P* value
Total patients	27,108(90.63)	2802 (9.37)	
PPI			**< 0.001**
PPI-Unexposed	24,752 (91.309%)	2377 (84.832%)	
PPI-Exposed	2356 (8.691%)	425 (15.168%)	
Age			**< 0.001**
[Mean ± SE]	48.793 ± 0.104	55.901 ± 0.306	
Gender			**< 0.001**
Male	13,001 (47.960%)	1562 (55.746%)	
Female	14,107 (52.040%)	1240 (44.254%)	
Race			**< 0.001**
Mexican American	4158 (15.339%)	362 (12.919%)	
Other Hispanic	2780 (10.255%)	314 (11.206%)	
Non-Hispanic White	10,863 (40.073%)	1536 (54.818%)	
Non-Hispanic Black	6100 (22.503%)	367 (13.098%)	
Other	3207 (11.830%)	223 (7.959%)	
BMI			**< 0.001**
[Mean ± SE]	29.126 ± 0.042	30.479 ± 0.129	
Uric acid (umol/L)			**< 0.001**
[Mean ± SE]	323.385 ± 0.506	335.357 ± 1.661	
Marital status			**< 0.001**
Married	13,610 (50.207%)	1595 (56.924%)	
Unmarried NA	13,485 (49.745%) 13 (0.048%)	1205 (43.005%) 2 (0.071%)	
Vigorous recreational activities			**< 0.001**
Yes	6157 (22.713%)	417 (14.882%)	
No	20,949 (77.280%)	2385 (85.118%)	
Moderate recreational activities			**< 0.001**
Yes	10,997 (40.567%)	980 (34.975%)	
No	16,106 (59.414%)	1822 (65.025%)	
Education			**< 0.001**
Less than 11th grade	6531 (24.093%)	699 (24.946%)	
High school or equivalent	6208 (22.901%)	628 (22.413%)	
Some college or AA degree	7956 (29.349%)	902 (32.191%)	
College graduate or above NA	6387 (23.561%) 26 (0.096%)	571 (20.378%) 2 (0.071%)	
Annual family income			0.2
$0–$19 999	6303 (23.616%)	657 (23.770%)	
$20 000 to $44 999 $45 000 to $74 999	8468 (31.727%)	913 (33.032%)	
	4691 (17.576%)	503 (18.198%)	
≥$ 75 000	6268 (23.484%)	601 (21.744%)	
Other	960 (3.597%)	90 (3.256%)	
Hypertension			**< 0.001**
Yes	9349 (34.488%)	1409 (50.286%)	
No NA	17,725 (65.387%) 34 (0.125%)	1391 (49.643%) 2 (0.071%)	
Diabetes			**< 0.001**
Yes	3264 (12.041%)	627 (22.377%)	
No Borderline NA	23,227 (85.683%) 605 (2.232%) 12 (0.044%)	2082 (74.304%) 90 (3.212%) 3 (0.107%)	
Daily intake [Mean (SD)]			
Total energy (kcal)	2102.522 (1006.679)	2069.503 (971.331)	0.163
Protein (gm)	80.949(42.996)	78.516 (41.519)	**0.003**
Dietary fiber (gm)	16.784 (10.607)	16.339 (10.696)	**0.018**
Calcium (mg)	920.938 (587.275)	900.681 (573.319)	0.082
Phosphorus (mg)	1343.272 (685.686)	1312.803 (664.271)	**0.015**
Sodium (mg)	3455.095 (1850.871)	3418.346 (1839.262)	0.256
Potassium (mg)	2606.341 (1264.534)	2567.264 (1258.676)	0.08
Magnesium (mg) Zinc (mg) Caffeine (mg) Alcohol (gm) Moisture (gm)	295.747 (151.014) 11.125 (8.249) 146.677 (203.729) 10.236 (28.570) 2882.537 (1514.389)	284.661 (145.128) 10.890 (6.583) 167.776 (232.773) 7.130 (26.944) 2824.019 (1475.853)	**< 0.001** 0.326 **< 0.001** **< 0.001** **0.038**
Vitamin A (mcg)	604.152 (644.738)	605.089 (665.539)	0.4
Vitamin B6 (mg)	2.063 (1.687)	1.969 (1.421)	**0.002**
Vitamin C (mg)	84.205 (97.310)	77.083 (94.029)	**< 0.001**
Vitamin D (mcg)	4.545 (5.607)	4.509 (5.489)	0.552
Vitamin E (mg)	8.207 (6.555)	8.175 (6.541)	0.399
Vitamin K (mcg) Alpha-carotene (mcg) Beta-carotene (mcg)	112.318 (197.265) 392.143 (1154.788) 2217.829 (4398.177)	101.966 (137.407) 369.472 (1354.182) 1982.022 (4142.071)	0.063 **0.027** **< 0.001**

*SE* standard error

### Logistic regression analysis and stratified analysis

The results are summarized in Table S1 and Table 2. Multiple weighted logistic regression models indicated that the PPIs exposure group (use only one PPI, P1) had a significantly higher risk of nephrolithiasis than the PPIs non-exposure group (no PPI use, P0) in Model 1(OR 1.88, 95% CI 1.68–2.10, *P* < 0.001), Model 2 (OR 1.39, 95% CI 1.24–1.57, *P* < 0.001) and Model 3 (OR 1.24, 95% CI 1.10–1.39, *P* < 0.001).

**Table 2:** Multivariate analysis of kidney stones by the amount of PPI intake, NHANES 2007–2018

	Model 3		
	OR (95% CI)	*P* value	P for interaction
***Overall***			
P0	1.00		
P1	1.26 (1.12-1.42)	**<0.001**	
***Gender***			0.111
***Male***			
P0	1.00		
P1	1.17(0.99-1.38)	0.064	
***Female***			
P0	1.00		
P1	1.39 (1.17-1.64)	**<0.001**	
***Race***			0.857
***Mexican American***			
P0	1.00		
P1	1.15 (0.80-1.65)	0.460	
***Other Hispanic***			
P0	1.00		
P1	0.98 (0.65-1.47)	0.918	
***Non-Hispanic White***			
P0	1.00		
P1	1.29 (1.11-1.50)	**<0.001**	
***Non-Hispanic Black***			
P0	1.00		
P1	1.26 (0.90-1.76)	0.175	
***Other***			
P0	1.00		
P1	1.50 (0.95-2.37)	0.083	
***Education***			0.475
***Less than 11th grade***			
P0	1.00		
P1	1.44 (1.16-1.79)	**0.001**	
***High school or equivalent***			
P0	1.00		
P1	1.16 (0.90-1.48)	0.247	
***Some college or AA degree***			
P0	1.00		
P1	1.14 (0.92-1.42)	0.243	
***College graduate or above***			
P0	1.00		
P1	1.27 (0.96-1.67)	0.094	
***Annual family income***			0.604
***$0–$19 999***			
P0	1.00		
P1	1.39 (1.11-1.73)	**0.004**	
***$20 000 to $44 999***			
P0	1.00		
P1	1.30 (1.06-1.59)	**0.013**	
***$45 000 to $74 999***			
P0	1.00		
P1	1.25 (0.93-1.68)	0.136	
***≥$ 75 000***			
P0	1.00		
P1	1.18 (0.89-1.56)	0.253	
***Other***			
P0	1.00		
P1	0.70 (0.30-1.61)	0.399	

Model 3: adjusted for gender, age, race, BMI (body mass index), uric acid, marital status, vigorous and moderate recreational physical activity, education level, annual family income, hypertension, diabetes, energy, protein, water, dietary intakes of calcium, phosphate, sodium, potassium, magnesium, zinc, Alpha-carotene, Beta-carotene, dietary fiber, caffeine, alcohol, vitamins A, B6, C, D, E, and K

The amount of PPI intake: P0 = no PPI use; P1 = use only one PPI.

We noticed that the results in stratified analysis by gender, there were significant differences between P1 and P0 for female participants in Model 1 (OR 1.90, 95% CI 1.62–2.23, *P* < 0.001), Model 2 (OR 1.58, 95% CI 1.34–1.87 *P* < 0.001) and Model 3 (OR 1.36, 95% CI 1.15–1.62, *P* < 0.001). In stratified analysis by race, there were significant statistical differences between P1 and P0 for individuals with a Non-Hispanic White in Model 1 (OR 1.69, 95% CI 1.46–1.95, *P* < 0.001), Model 2 (OR 1.43, 95% CI 1.23–1.65 *P* < 0.001) and Model 3 (OR 1.27, 95% CI 1.09–1.48, *P* = 0.002). In stratified analysis by education, there were significant statistical differences between P1 and P0 for individuals with a less than 11th grade in Model 1 (OR 2.02, 95% CI 1.65–2.48, *P* < 0.001), Model 2 (OR 1.56, 95% CI 1.26–1.93 *P* < 0.001) and Model 3 (OR 1.41, 95% CI 1.13–1.76, *P* = 0.002). In stratified analysis by annual family income, there were significant statistical differences between P1 and P0 for individuals with an income of $0 to $19,999 and $20,000 to $44,999 in three models. The study found no significant correlation between PPI and kidney stones across all demographic groups, including gender, race, education, and annual family income (P for interaction > 0.05).

## Discussion

The results of our study indicated that the use of PPIs was associated with a higher prevalence of kidney stones. Furthermore, the stratified analysis revealed significant statistical differences between PPIs use and kidney stones among females, non-Hispanic whites, individuals with low education levels and individuals with low household income levels.

The pathogenesis of kidney stones is very complex and varies according to different stone components. The abnormal composition of urine leading to the formation of stone salt crystals is one of the factors [25]. The use of PPIs may affect urinary calcium, citrate and magnesium, which may further contribute to the formation of kidney stones [15–24]. In a conference abstract, the authors used data from the Electronic Health Record (EHR) to find that in patients with no history of kidney stones, 24-hour urinary magnesium and urinary citrate were lower in the PPIs exposure group than in the non-PPIs exposure group [26]. Prior to this article, a cohort study on the Women’s Veterans Cohort Study (WVCS) found that PPIs use was associated with an increased incidence of kidney stones [27]. Previously, PPIs use was associated with kidney injury, electrolyte abnormalities, and kidney stones using FDA adverse event data [28]. No researchers have analyzed kidney stones and PPIs use through the NHANES database, and previous studies have been partial to blaming abnormal urine composition for the development of kidney stones.

CaOx kidney stones are the most common kidney stone, and their origin is closely related to RP [3, 31]. Furthermore, oxidative stress and inflammatory response caused by calcium phosphate (CaP) deposition in the renal papilla can accelerate the growth of RP [32]. Fontecha-Barriuso et al. found that omeprazole increased renal tubular cell death in mice and the expression of NGAL and HO-1, both markers of kidney damage and oxidative stress, and the kidneys of PPIs drugs toxicity may be related to oxidative stress [33]. The occurrence of CaOx stones may be related to the abnormal oxidative stress induced by PPIs, which needs to be proved by specific laboratory studies on kidney stones and PPIs. Moreover, all databases used in cross-sectional studies on kidney stones and PPIs lack kidney stones components, which is a loss that cannot be ignored. In a randomized controlled trial, there were differences between the diurnal variation in urine acidification of normal individuals and uric acid stone formers, and PPIs use did not affect this change [34]. Stone composition is an essential part of relevant research, which may help reveal the mechanism of PPIs on the occurrence of kidney stones with different components.

We found an association between kidney stones and PPIs use in females. GERD is an indication for PPIs, and females are more likely to have persistent symptoms of GERD [35]. Furthermore, a clinical study found that the Cmax, half-life and elimination half-life of omeprazole were significantly increased in women compared with men [36]. These may indicate that women are more likely to take PPIs for treatment and that PPIs drug metabolism may be slower in women than in men. CYP2C19 is a cytochrome that affects PPIs metabolism, and its activity can be decreased by oral contraceptives containing acetylene estradiol, which may reflect the inhibitory effect of estrogen on PPIs drug metabolism [37, 38]. However, studies have found that estrogen and estrogen receptor signaling pathways may inhibit renal cell damage caused by oxidative stress [39, 40]. Moreover, estrogen receptor β signaling may inhibit renal CaOx crystal deposition by reducing oxidative stress in renal tubular cells [41]. Although female individuals are less likely to develop kidney stones than male individuals, the suppression of PPIs drug metabolism under the influence of estrogen may lead to more extensive kidney damage, thus increasing the risk of developing kidney stones.

We also found that PPIs use was associated with an increased incidence of kidney stones in non-Hispanic white individuals. In a retrospective analysis, PPIs healing rates were inconsistent between nonwhites and whites in the treatment of erosive oesophagitis [42]. Additionally, the distribution of variant alleles of CYP2C19, which is related to PPIs metabolism, is significantly different among races, and this variant allele can cause the deletion of some functional genes [37]. In a previous NHANE cross-section study, non-Hispanic whites were associated with a higher incidence of kidney stones [43]. The effects of PPIs may vary among ethnic groups, and the incidence of kidney stones may be ethnically related. Whereas, only cross-sectional studies are available for reference, which requires more longitudinal studies to verify this relationship.

There are several limitations to our study. First, because our study design used a cross-sectional study, it is difficult to determine a causal relationship between PPIs and kidney stones in our results. Second, there may be unknown confounding factors influencing the study results. Third, the NHANES database reports only prescription drug use, and there may be participants taking PPIs without a prescription. Fourth, the cumulative dose of PPI cannot be obtained in the NHANES database, and further dose-stratified causal correlation analysis cannot be performed. Fifth, there is a significant amount of missing data related to the number of minutes of vigorous and moderate exercise per day in NHANES. Out of the 29,910 individuals included in our analysis, more than 25,000 are missing this information. We are unable to accurately classify and analyze the specific exercise time. Sixth, there is no information on stone composition that may further illuminate the relationship between PPIs and kidney stones. Finally, our study needs to be validated by more longitudinal and laboratory studies to elucidate the mechanism of PPIs and the occurrence of kidney stones.

## Conclusions

We found that PPIs use may be associated with a higher prevalence of kidney stones, primarily among women, non-Hispanic whites, individuals with low education levels and individuals with low household income levels. Further studies are required to confirm our findings and clarified the biological mechanisms.

### Electronic supplementary material

Below is the link to the electronic supplementary material.

Supplementary Material 1

## Data Availability

The datasets generated during and/or analysed during the current study are available in the NHANES repository, https://wwwn.cdc.gov/nchs/nhanes/continuousnhanes/default.aspx?BeginYear=2007, https://wwwn.cdc.gov/nchs/nhanes/continuousnhanes/default.aspx?BeginYear=2009, https://wwwn.cdc.gov/nchs/nhanes/continuousnhanes/default.aspx?BeginYear=2011, https://wwwn.cdc.gov/nchs/nhanes/continuousnhanes/default.aspx?BeginYear=2013, https://wwwn.cdc.gov/nchs/nhanes/continuousnhanes/default.aspx?BeginYear=2015, https://wwwn.cdc.gov/nchs/nhanes/continuousnhanes/default.aspx?BeginYear=201.

## Abbreviations

CaOx: Calcium oxalate
RP: Randall’s plaque
PPIs: proton pump inhibitors
GERD: gastroesophageal reflux disease
PUD: peptic ulcer disease
OGTT: oral glucose tolerance test
NHANES: National Health and Nutrition Survey
BMI: body mass index
SE: standard error
OR: odds ratio
CI: confidence interval
MEC: mobile examination center
P0: PPI non-exposure group
P1: PPI exposure group
HER: Electronic Health Record
WVCS: Women’s Veterans Cohort Study
Cmax: peak concentration
CaP: calcium phosphate
CYP: cytochromes P450
