# Supplementary material for: Proton pump inhibitors use is associated with a higher prevalence of kidney stones: NHANES 2007–2018
Source: BMC Public Health. 2024 May 2;24:1215. doi: 10.1186/s12889-024-18710-8 (PMC11067170; doi:10.1186/s12889-024-18710-8)
Supplement: Supplementary file 1 — Supplementary Material 1 [file 12889_2024_18710_MOESM1_ESM.docx]

**Table S1** Multivariate analysis of kidney stones by the amount of PPI intake, NHANES 2007–2018.

|  | Model 1 | |  | Model 2 | |  |
| --- | --- | --- | --- | --- | --- | --- |
|  | OR (95% CI) | P value |  | OR (95% CI) | P value |  |
| ***Overall*** |  |  |  |  |  |  |
| P0 | 1.00 | |  | 1.00 | |  |
| P1 | 1.88 (1.68-2.10) | **<0.001** |  | 1.39 (1.24-1.57) | **<0.001** |  |
| ***Gender*** |  |  |  |  |  |  |
| ***Male*** |  |  |  |  |  |  |
| P0 | 1.00 | |  | 1.00 | |  |
| P1 | 1.93 (1.65-2.26) | **<0.001** |  | 1.26 (1.07-1.49) | **0.005** |  |
| ***Female*** |  |  |  |  |  |  |
| P0 | 1.00 | |  | 1.00 | |  |
| P1 | 1.90 (1.62-2.23) | **<0.001** |  | 1.58 (1.34-1.87) | **<0.001** |  |
| ***Race*** |  |  |  |  |  |  |
| ***Mexican American*** |  |  |  |  |  |  |
| P0 | 1.00 | |  | 1.00 | |  |
| P1 | 1.92 (1.36-2.71) | **<0.001** |  | 1.29 (0.90-1.84) | 0.169 |  |
| ***Other Hispanic*** |  |  |  |  |  |  |
| P0 | 1.00 | |  | 1.00 | |  |
| P1  ***Non-Hispanic White***  P0  P1  ***Non-Hispanic Black***  P0  P1  ***Other***  P0  P1  ***Education***  ***Less than 11th grade***  P0  P1  ***High school or equivalent***  P0  P1  ***Some college or AA degree***  P0  P1  ***College graduate or above***  P0  P1  ***Annual family income***  ***$0–$19 999***  P0  P1  ***$20 000 to $44 999***  P0  P1  ***$45 000 to $74 999***  P0  P1  ***≥$ 75 000***  P0  P1  ***Other***  P0  P1 | 1.39 (0.95-2.03)  1.00  1.69 (1.46-1.95)  1.00  1.81 (1.31-2.49)  1.00  2.54 (1.65-3.91)  1.00  2.02 (1.65-2.48)  1.00  1.72 (1.36-2.17)  1.00  1.74 (1.42-2.15)  1.00  2.07 (1.59-2.69)  1.00  1.96 (1.59-2.41)  1.00  2.00 (1.65-2.43)  1.00  1.85 (1.40-2.43)  1.00  1.74 (1.33-2.27)  1.00  1.22 (0.57-2.63) | 0.094  **<0.001**  **<0.001**  **<0.001**  **<0.001**  **<0.001**  **<0.001**  **<0.001**  **<0.001**  **<0.001**  **<0.001**  **<0.001**  0.608 |  | 1.09 (0.74-1.63)  1.00  1.43 (1.23-1.65)  1.00  1.36 (0.98-1.89)  1.00  1.86 (1.19-2.92)  1.00  1.56 (1.26-1.93)  1.00  1.24 (0.98-1.59)  1.00  1.32 (1.06-1.64)  1.00  1.41 (1.07-1.85)  1.00  1.54 (1.24-1.91)  1.00  1.44 (1.18-1.76)  1.00  1.33 (1.00-1.77)  1.00  1.29 (0.98-1.69)  1.00  0.92 (0.42-2.02) | 0.659  **<0.001**  0.070  **0.006**  **<0.001**  0.079  **0.012**  **0.013**  **<0.001**  **<0.001**  0.053  0.075  0.827 |  |
|  |  |  |  |  |  |  |

Model 1: no covariates were adjusted.

Model 2: adjusted for gender, age and race.

The amount of PPI intake: P0 = no PPI use; P1 = use only one PPI.
